# Muscle Contraction Induces Acute Hydroxymethylation of the Exercise-Responsive Gene *Nr4a3*

**DOI:** 10.3389/fendo.2016.00165

**Published:** 2016-12-23

**Authors:** Pattarawan Pattamaprapanont, Christian Garde, Odile Fabre, Romain Barrès

**Affiliations:** ^1^Department of Physiology, Faculty of Science, Mahidol University, Bangkok, Thailand; ^2^Novo Nordisk Foundation Center for Basic Metabolic Research, Faculty of Health and Medical Sciences, University of Copenhagen, Copenhagen, Denmark

**Keywords:** skeletal muscle, muscle contraction, gene expression regulation, epigenetics, DNA methylation

## Abstract

Exercise training triggers numerous positive adaptations through the regulation of genes controlling muscle structure and function. Epigenetic modifications, including DNA methylation, participate in transcriptional activation by allowing the recruitment of the transcription machinery to gene promoters. Exercise induces dynamic DNA demethylation at gene promoters; however, the contribution of the demethylation precursor hydroxymethylcytosine is unknown. Given the evanescent nature of hydroxymethylcytosine, a muscle contraction model that allows for the collection of samples that are repeatedly stimulated over time is required to determine whether contraction-induced demethylation is preceded by changes in the hydroxymethylcytosine level. Here, we established an acute skeletal muscle contraction model to mimic the effects of acute exercise on gene expression. We used this model to investigate the effect of muscle contraction on DNA demethylation and hydroxymethylation. First, we performed an acute exercise study in healthy humans to identify an exercise-responsive gene that we could study in culture. We identified the nuclear receptor subfamily 4 group A member 3 (*Nr4a3*) gene with the highest fold-expression increase after acute exercise. We then refined an electrical pulse stimulation (EPS) protocol that could induce expression of the *Nr4a3* gene in C2C12 myotubes. Using targeted bisulfite sequencing, we found that in response to EPS, a region of the *Nr4a3* promoter is rapidly demethylated at 60 min and re-methylated at 120 min. Of interest, hydroxymethylation of the differentially methylated region of *Nr4a3* promoter after EPS was elevated immediately after EPS, with lowest levels reached at 60 min after EPS. In conclusion, we have established a cell culture-based protocol to mimic the acute transcriptional responses to exercise. Furthermore, we provide insight into the mechanism by which the exercise-responsive gene *Nr4a3* is demethylated after muscle contraction.

## Introduction

Physical exercise remodels skeletal muscle structure and function through the regulation of numerous genes controlling muscle metabolism, structure, and growth (1). At the DNA level, the regulation of gene expression is orchestrated by epigenetic mechanisms that coordinate the recruitment of transcription and *cis*-regulatory factors to gene promoters (2). DNA methylation and histone acetylation constitute the two major epigenetic modifications implicated in exercise-induced gene expression. Indeed, following an acute exercise bout, histone deacetylases 4 and 5 are exported to the nuclei of the muscle fiber, which allows increased acetylation of histone proteins and subsequent relaxation of chromatin at exercise-responsive loci (3). More recently, we have shown that muscle contraction induces dramatic demethylation at promoter regions of peroxisome proliferator-activated receptor delta (*PPARD*), pyruvate dehydrogenase lipoamide kinase isozyme 4 (*PDK4*), and peroxisome proliferator-activated receptor gamma coactivator 1-alpha (*PPARGC1A*) (4), suggesting that targeted DNA demethylation facilitates the initiation of the transcription machinery at exercise-responsive genes.

Active DNA demethylation occurs in non-dividing cell through a pathway involving the ten-eleven translocation (TET) enzymes, which catalyze several steps of the demethylation process, notably by the generation of hydroxymethyl cytosine, an intermediate for demethylation (5). To date, the effect of muscle contraction on hydroxymethylation at exercise-responsive genes is unknown. The fugacity of hydroxymethylation intermediate in the DNA demethylation process is likely to account for this lack of evidence. To address this question, a cell-based model amenable to the collection of data points at short intervals is required. Several *ex vivo* or *in vitro* models exist to study muscle contraction. Cell culture models represent the “*in vitro*” models of choice for time series investigations. Various cell culture models using electrical stimulation have been proposed (6–13); however, these models mimic long-term contraction and are aiming at inducing changes in protein abundance. Thus, they are not suitable for investigating the acute effect of muscle cell (myotube) contraction on mRNA expression.

In this study, we established an acute skeletal muscle contraction model that partly mimics the effect of exercise on gene expression. We used this model to investigate the effect of muscle contraction on DNA methylation remodeling. We refined an electrical pulse stimulation (EPS) protocol in differentiated C2C12 myotubes that we benchmarked against changes in the expression of nuclear receptor subfamily 4 group A member 3 (*Nr4a3*), a gene selected based on our observation that it shows the highest fold increase in expression after acute exercise in untrained humans. Using this assay, we provide evidence that the *Nr4a3* promoter is rapidly hydroxymethylated prior to demethylation, supporting a role for hydroxymethylation in contraction-induced DNA methylation remodeling in skeletal muscle.

## Materials and Methods

### Acute Exercise Study

Ten healthy Danish male volunteers, aged between 19 and 25 (22.6 ± 1.6) years old, were recruited to participate in the study. The participants were sedentary and had a body mass index between 19.5 and 28.5 (23.1 ± 2.8, more clinical characteristics can be found in Table S1 in Supplementary Material). All participants reported to the laboratory and completed a peak pulmonary oxygen uptake test under fasting conditions to determine their VO_2_ max. This test consisted of an incremental exercise bout to volitional fatigue on an electromagnetically braked cycle ergometer (Monark Ergomedic 839E) during which pulmonary gas exchange was measured breath-by-breath with a gas analyzing system (Oxycon Pro, Jaeger). The participants then performed an intense (80% VO_2_ max) 15-min exercise bout and skeletal muscle biopsies were collected under fasting conditions at rest (basal) and 4 h after cessation of exercise. The aerobic (rather than resistance) exercise was chosen since the literature reporting EPS model describe genes that respond to aerobic exercise (6, 12). The 4-h time point was chosen based on previous observations from our group and others, which peaked that mRNA expression is seen 3–5 h after exercise (1, 4). Biopsies were obtained from the *Vastus lateralis* muscle using the Bergström needle technique (14), and they were immediately snap-frozen in liquid nitrogen and stored at −80°C for further analysis.

This study was carried out in accordance with the recommendations of the Ethic Committee from the Capital Region of Denmark with written informed consent from all subjects. All subjects gave written informed consent in accordance with the Declaration of Helsinki. The protocol was approved by the Ethic Committee from the Capital Region of Denmark (reference H-1-2013-064).

### Total RNA Purification

Total RNA was purified from the skeletal muscle biopsy samples using an AllPrep DNA/RNA/miRNA Universal Kit (Qiagen) following manufacturer’s instructions. The quality of recovered RNA was assessed by using the Agilent RNA 6000 Nano Kit (Agilent Technologies). RNA concentration was determined by spectrophotometry using a NanoDrop 2000. After purification, RNA samples were stored at −80°C until further analysis.

### RNA Sequencing

Libraries were prepared after ribosomal RNA depletion with the TruSeq Stranded Total Sample Prep Kit with Ribo-Zero Gold (Illumina). Sequencing was done on a HiSeq 2500, single-read, 100 bp read length per lane. Reads were trimmed for adapters and low quality flanking ends using Trim Galore v0.3.7 and Cutadapt v1.4.2 (15). Pre-processed reads were mapped to the human reference genome (hg19) with tophat2 using the “--b2-very-sensitive” setting (16, 17), and gene coverages were computed with featureCounts (18) using the Gencode v19 annotation. Genes with a coverage larger than 2.5 RPKM in at least five samples were tested for differential expression with edgeR using the glmQLFit/glmQLFTest modeling framework (19).

### Cell Culture

Mouse skeletal muscle cell line C2C12 (ATCC CRL1772) were seeded into six-well plates at a density of 10^5^ cells/well, cultured in growth medium containing Dulbecco’s modified Eagle’s medium (25 mM glucose) supplemented with 10% fetal bovine serum (FBS), and 1% penicillin–streptomycin at 37°C, 5% CO_2_. After reaching ~90% confluence, differentiation was induced by switching 10% FBS to 2% horse serum. Differentiation medium was changed every day until fully differentiated myotubes were visualized under a light microscope (5–6 days).

### Electrical Pulse Stimulation

**Figure 1:**
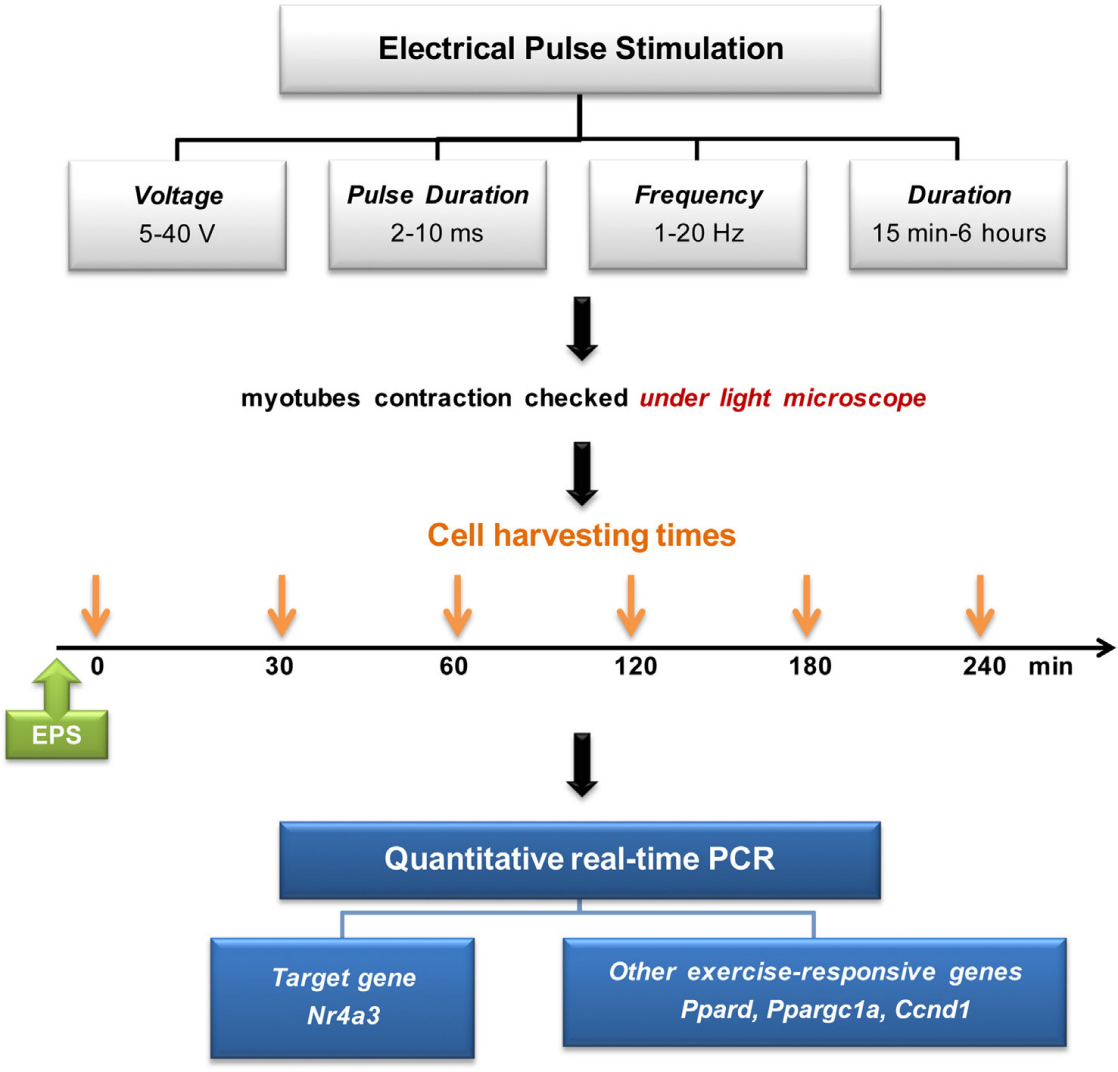
**Electrical pulse stimulation setup and experimental design**.

A six-well plate, containing differentiated C2C12 myotubes was connected to C-dish electrode (IonOptix). The electrical current was discharged from a carbon electrode that was immersed in 8 ml differentiation medium added 24 h before the EPS. EPS was applied by C-pace cell culture stimulator (C-pace EP, IonOptix). EPS-induced muscle contraction was validated using light microscopy. Voltage, frequency, pulse duration, and stimulation time were optimized to induce reproducible increases in mRNA expression of exercise-responsive genes (Figure 1). Cells were harvested immediately after EPS at 0, 30, 60, 120, 180, and 240 min.

### Gene Expression Analysis

Total RNA was isolated from cells with TRIzol (Invitrogen) or AllPrep DNA/RNA/miRNA universal kit (Qiagen) according to the manufacturer’s instructions. RNA was converted to cDNA by iScript cDNA synthesis kit (Bio-Rad). Gene expression was determined by quantitative real-time PCR (qPCR) using Brilliant III Ultra-Fast SYBR Green Master mix (Agilent Technologies). Sequences of qPCR primers used for *Ppargc1a, Ppard, Cyclin D1* (*Ccnd1*), and *Nr4a3* genes are provided (Table S2 in Supplementary Material). mRNA expression was quantified by normalizing Ct values to cDNA amounts.

### DNA Isolation

Genomic DNA was isolated from the muscle cell cultures using an AllPrep DNA/RNA/miRNA Universal kit (Qiagen) according to the manufacturer’s instructions.

### DNA Methylation Analysis

Genomic DNA was bisulfite-converted using EZ DNA Methylation-Lightning Kit (Zymo Research) according to the manufacturer’s instructions. Bisulfite primers were designed to span the promoter region of *Ppard, Ccnd1*, and *Nr4a3* genes within −3,000 to +2,000 bp relative to transcriptional start site (TSS) (Table S2 in Supplementary Material) using MethPrimer program (20). All bisulfite primers were mixed at equimolar *ratio* in a multiplexed PCR reaction using 200 ng of DNA as starting material and the HotStarTaq Plus DNA Polymerase kit (Qiagen). The PCR program was run at 95°C for 5 min, 35 cycles of 94°C (1 min) 60°C (1 min) 72°C (1 min), followed by 72°C for 10 min. PCR products were purified using MinElute PCR purification Kit (Qiagen). DNA libraries were prepared using NEBNext Ultra DNA Library Prep Kit for Illumina (New England Biolabs) and sequenced on a Miseq instrument (Illumina) with 151 cycles paired-end run. After sequencing, reads were trimmed for adapters and low quality flanking ends using Trim Galore v0.3.7 and Cutadapt v1.4.2 (15). Pre-processed reads were mapped to the mm10 reference genome and DNA methylation levels quantified by Bismark v0.14.4 and Bowtie2 (21, 22).

### DNA Hydroxymethylation Analysis

DNA (1 µg) was sheared into fragments (~200–300 bp) using a Bioruptor Plus sonicator (Diagenode). Hydroxymethylated DNA was captured from 220 ng of fragmented DNA using the hydroxymethylated DNA Immunoprecipitation (hMeDIP) ChIP Kit (Abcam) according to the manufacturer’s instructions. PCR primers were designed to cover a region of *Nr4a3* promoter predetermined by DNA methylation analysis in response to EPS (Table S2 in Supplementary Material). DNA hydroxymethylation levels were determined by qPCR using Brilliant III Ultra-Fast SYBR Green Master mix (Agilent Technologies). Hydroxymethylated DNA was quantified by normalizing to total DNA input.

### Data Analysis

Data are presented as mean ± SE. Statistical analysis was performed using Graphpad Prism 6 (GraphPad Software). Two-way ANOVA was performed to determine the effect of EPS on gene expression, DNA methylation, and hydroxymethylation. When differences were statistically significant, the difference between control (non-stimulated) and EPS groups at corresponding time points was compared by an unpaired *t*-test.

## Results

### Identification of Exercise-Responsive Genes in Humans

We performed RNA sequencing on human skeletal muscle biopsies collected before and 4 h after an acute exercise bout to identify genes showing the highest transcriptional response. Analysis of differential mRNA expression showed 165 differentially expressed genes (out of 6,242 genes detected), where 72 were over-expressed and 93 were under-expressed after acute exercise (Table 1; Table S3 in Supplementary Material). The most upregulated gene was the Nuclear Receptor subfamily 4 group A member 3 (*NR4A3*) (*P* = 3.41E−08; false discovery rate = 2.51E − 05), which increased 26-fold after acute exercise. Thus, we selected *NR4A3* to benchmark a model of muscle contraction in cultured myotubes.

**Table 1:** **Genes showing highest fold upregulation after acute exercise in skeletal muscle from young healthy humans**.

Gene name	RefSeq. ID	Fold change	*P*-value	FDR
*NR4A3*	NM_173199	26.0	3.41E−08	2.51E−05
*PRKAG2*	NM_016203	6.1	5.93E−13	6.98E−09
*RTN4RL1*	NM_178568	4.3	3.08E−09	3.30E−06
*WNT9A*	NM_003395	4.2	1.09E−09	1.60E−06
*CHST15*	NM_015892	3.9	1.06E−10	2.09E−07
*FCN3*	NM_003665	3.7	1.80E−06	5.04E−04
*FKBP5*	NM_004117	3.6	4.48E−08	2.93E−05
*KCNQ4*	NM_004700	3.3	4.95E−08	3.07E−05
*PMP22*	NM_153321	3.2	5.80E−11	2.09E−07
*METTL7B*	NM_152637	3.0	0.0015	0.0801

*The top 10 genes are showed. The complete list can be found in Table S3 in Supplementary Material*.

*FDR, false discovery rate*.

### EPS Mimics the Effect of Exercise on *Nr4a3*

**Figure 2:**
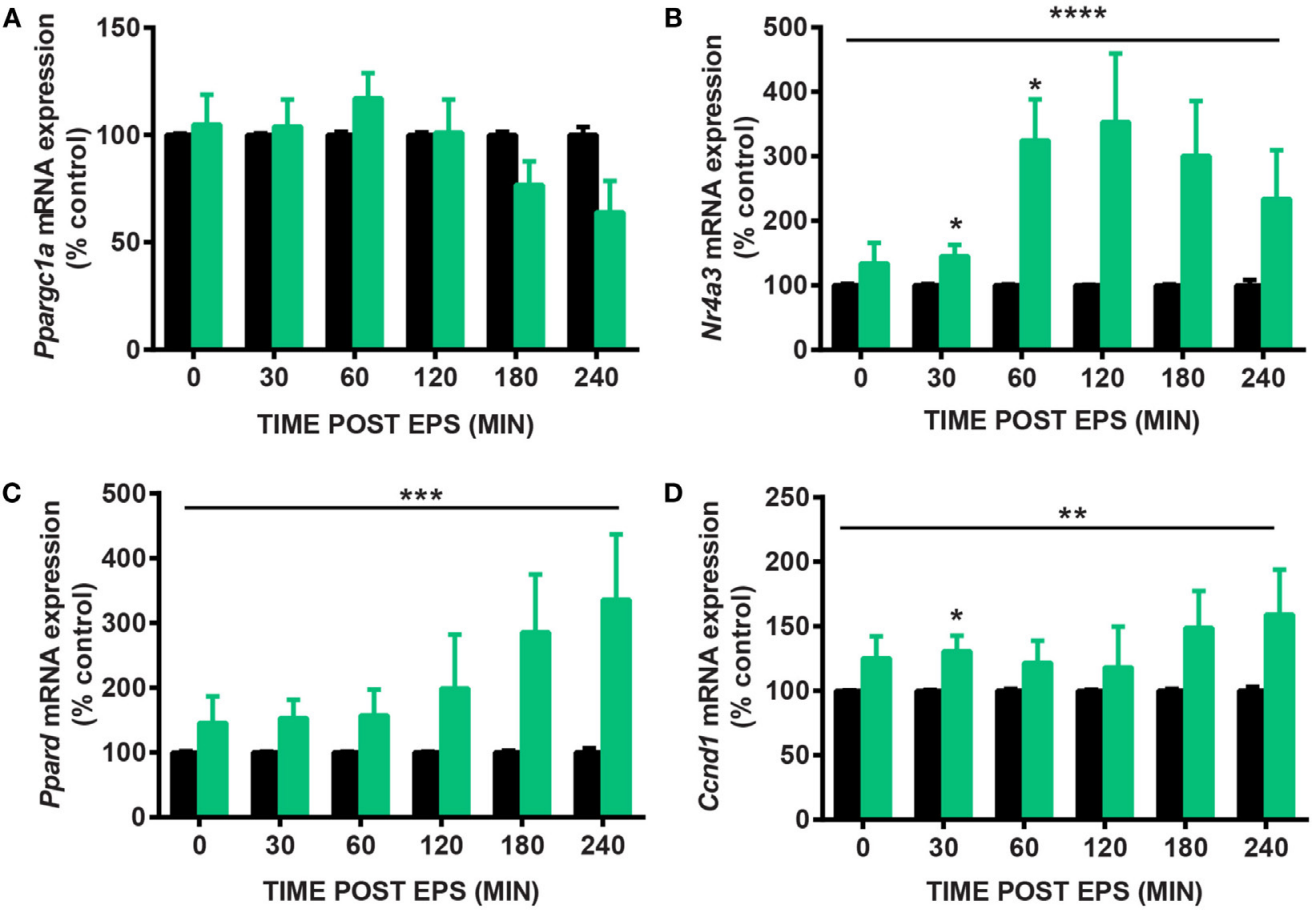
**Gene expression changes after electrical pulse stimulation (EPS) contraction**. C2C12 myotubes were subjected to EPS at 2 ms, 30 V, and 1 Hz for 30 min and harvested immediately (0), 30, 60, 120, 180, and 240 min after EPS. Quantitative real-time PCR measures of *Ppargc1a* **(A)**, *Nr4a3* **(B)**, *Ppard* **(C)**, and *Ccnd1* **(D)** are shown for unstimulated cells (CON, black bars) and EPS-stimulated cells (EPS, green bars). For each time point, EPS value is expressed as percentage of control value which is set at 100%. Data are presented for four independent experiments as mean ± SE (*P < 0.05, **P < 0.01, ****P < 0.0001 EPS-stimulated vs. control).

Electrical pulse stimulation of cultured myotubes has been described (9, 11) and used to potentiate myotube differentiation (8) and increase the expression of specific genes, notably *Ppargc1a* (6). Here, we developed a novel EPS protocol to mimic the effects of acute exercise on the expression of *Nr4a3*. We tested several EPS conditions to stimulate C2C12 myotubes with contraction (Figure 1). While an EPS protocol applying 2 ms, 30 V, and 1 Hz had no effect on *Ppargc1a* mRNA expression (Figure 2A), it consistently induced the expression of *Nr4a3* up to threefold at 60 min (Figure 2B) and also led to changes in mRNA expression of *Ppard* and *Ccnd1* (Figures 2C,D). Thus, we selected this EPS protocol to study the epigenetic response of *Nr4a3, Ccnd1*, and *Ppard* after acute contraction.

### EPS Induces Transient *Nr4a3* Promoter Demethylation

**Figure 3:**
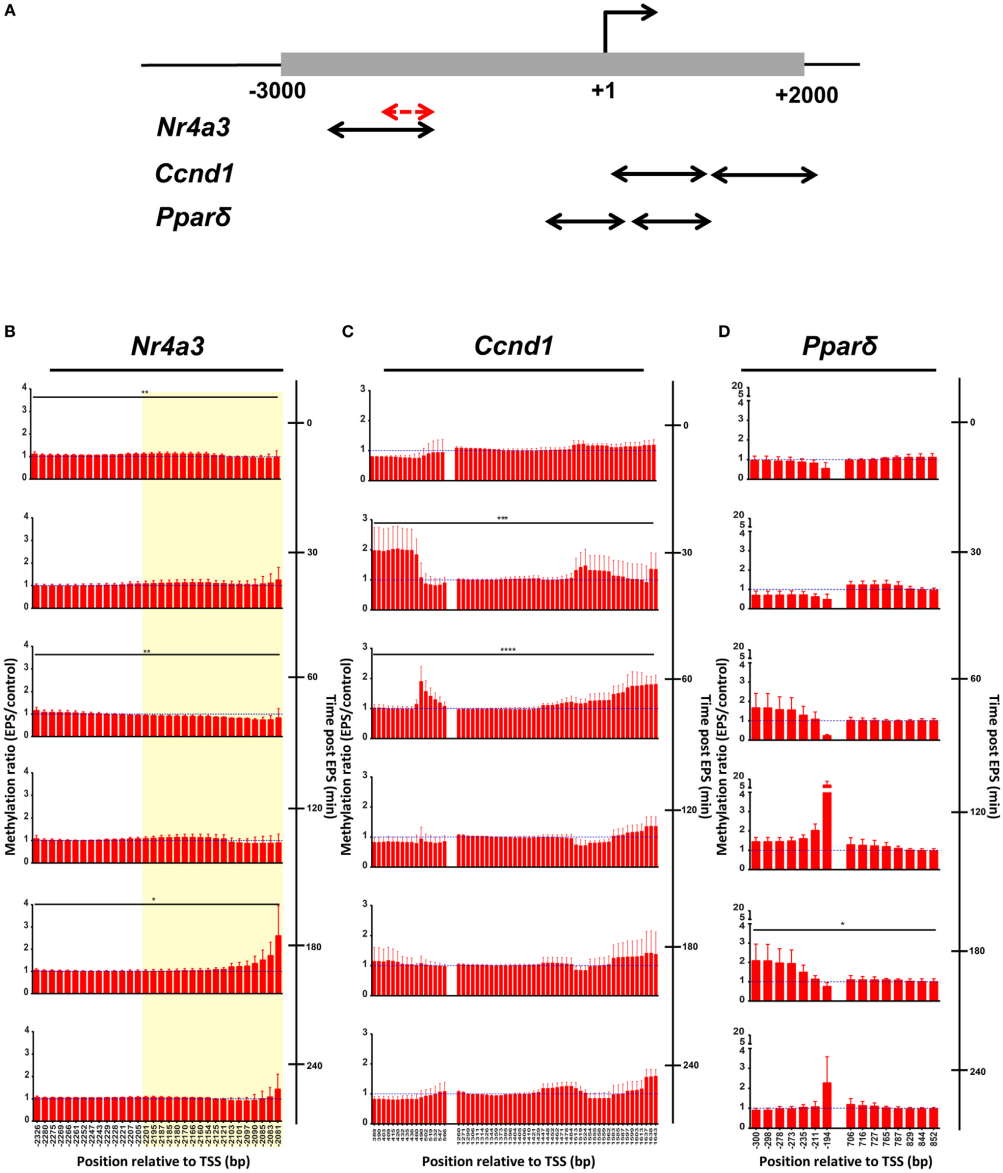
**DNA methylation is remodeled by electrical pulse stimulation (EPS) contraction**. **(A)** Schematic view of regions analyzed by bisulfite sequencing. One to two pairs of primers were designed for each gene. All primers were located within −3,000 to +2,000 bp relative to transcriptional start site (black arrows). A fraction of *Nr4a3* promoter was further selected for hydroxymethylation analysis in Figure 4 (red arrows) and highlighted in yellow area on the *Nr4a3* graph **(B)**. DNA methylation of *Nr4a3* **(B)**, *Ccnd1* **(C)**, and *Ppard* **(D)**. Methylation ratios between the unstimulated control cells and EPS-stimulated at various time points are shown. For each time point, ratio of 1 is indicated by a blue, hatched line. Data are presented for three independent experiments as mean ± SE (*P < 0.05, **P < 0.01, ***P < 0.001, ****P < 0.0001 EPS-stimulated vs. control).

We used targeted bisulfite sequencing to investigate the effect of EPS on *Nr4a3, Ccnd1*, and *Ppard* methylation. EPS altered the methylation status of several cytosines located in a region encompassing −2,201 to −2,081 bp relative to the TSS of the *Nr4a3* gene (Figure 3A). Methylation of this region was inversely associated with *Nr4a3* mRNA expression measured 60 min after EPS, at a time point where methylation was decreased (Figures 2B and 3B). Interestingly, *Nr4a3* methylation was increased at 180 min, which marks the time point where *Nr4a3* mRNA expression started to decrease (Figures 2B and 3B). EPS-induced transient methylation of the region +389 to +566 bp relative the TSS of the *Ccnd1* gene at 30 and 60 min post-EPS (Figure 3C), which was associated with elevated mRNA expression at the 30-min time point (Figure 2D), suggesting hypermethylation is secondary to gene expression. Of potential interest, a wave of demethylation on the *Ccnd1* promoter was detected at 120 min post-EPS, with mRNA expression tending to increase at the following two time points (Figure 2D). Similarly, methylation level of the *Ppard* promoter at a region of +706 to +852 bp relative to TSS was decreased at 60 min post-EPS (Figure 3D), which was followed by a progressive increase in *Ppard* mRNA expression in the following time points (Figure 2C). Our results provide evidence that EPS induces DNA methylation remodeling. Furthermore, we also show that contraction-induced DNA demethylation precedes transcriptional activation.

### EPS Rapidly Increases Hydroxymethylation of the *Nr4a3* Promoter

**Figure 4:**
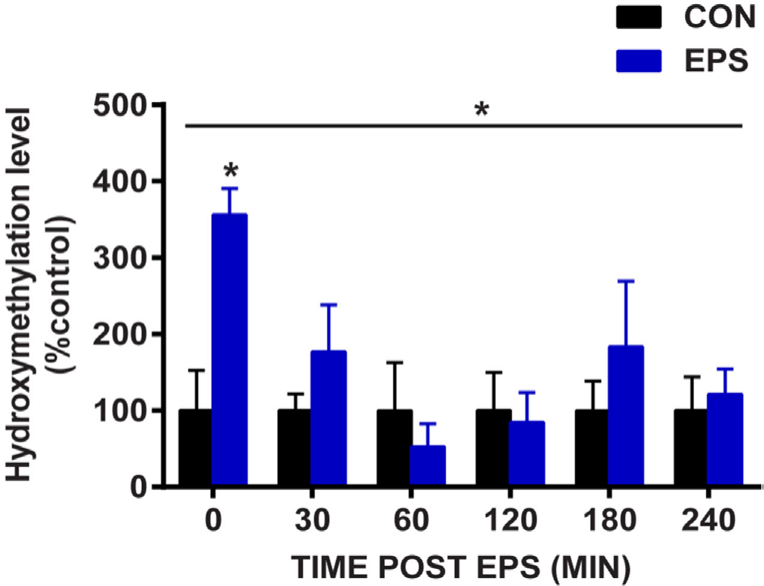
**Electrical pulse stimulation (EPS) increases hydroxymethylcytosine levels in *Nr4a3* promoter**. DNA from unstimulated cells (CON, black bars) or subjected to EPS (EPS, blue bars) was analyzed for hydroxymethylation at a specific region of *Nr4a3* (schematized in Figure 3A, red arrows) using hydroxymethylcytosine capture followed quantitative real-time PCR. For each time point, EPS value is expressed as percentage of control value which is set at 100%. Data for three independent experiments are presented as mean ± SE (*P < 0.05 EPS-stimulated vs. control).

Given that EPS induced robust change in mRNA expression and demethylation of the *Nr4a3* gene, we investigated the amount of the demethylation precursor hydroxymethylcytosine in the region −2,201 to −2,081 bp relative to the TSS of *Nr4a3*. We used hydroxymethylcytosine capture followed by quantitative PCR to gain further insight into the potential mechanisms by which EPS-induced myotube contraction induces *Nr4a3* demethylation. We detected a threefold increase in hydroxymethylation levels of *Nr4a3* immediately after EPS-induced contraction (0 min time point, Figure 4). This increase was transient, with the hydroxymethylation level restored to the baseline level by 30 min and further decreased at the 60 and 120 min time points (Figure 4). As the methylation level corresponding to this region was decreased at 60 and 120 min (Figure 3A), our results strongly suggest that contraction-induced *Nr4a3* promoter demethylation occurs through intermediate hydroxymethylation.

## Discussion

We identified *NR4A3* as an exercise-responsive gene showing the highest fold change in mRNA expression using RNASeq of skeletal muscle biopsies collected from healthy untrained men after acute exercise. The *Nr4a3* gene has previously been associated with enhanced metabolic function and endurance capacity (23). Consistent with our RNASeq data, *NR4A3* has previously been reported to show the highest fold change in mRNA level in response to acute exercise (24, 25). In addition, *Nr4a3* expression is increased in mouse skeletal muscle immediately after exercise or in response to two different types of exercise in combination with electrical stimulation (26). Based on these data, we established an EPS-based model of muscle contraction to mimic the effect of muscle contraction on the *Nr4a3* gene.

Using an EPS protocol mimicking the effect of exercise in cultured muscle cells, we performed time course experiments and quantified gene expression of exercise-responsive genes. EPS altered mRNA level of *Ccnd1* and *Ppard*, two genes respectively involved in muscle growth and metabolism and previously shown to be induced by muscle contraction (27, 28). In contrast, our EPS protocol failed to induce *Ppargc1a* mRNA expression although several studies including one from our group have reported that *Ppargc1a* expression is increased after a single bout of exercise (4, 25, 28, 29). The lack of response for *Ppargc1a* gene in our hands is likely due to the nature of the contraction protocol, as previous reports show *Ppargc1a* gene expression is induced by slow-type tetanic contraction (30). Conversely, an EPS protocol of 1 ms, 14 V, and 50 Hz at 1-s trains/1-s pauses on C2C12 induced *Ppargc1a* gene expression after 24 h, but not in cells stimulated for less than 90 min (6). Likewise, EPS contraction at 2 ms, 14 V, and 5 Hz of primary human myotubes induced *PPARGC1A* gene expression after 24 h, but failed to elicit changes after 2, 4, or 8 h of stimulation (12). Collectively, these data suggest that our EPS model can mimic some, but not all the features of muscle contraction *in vivo*. As exercise triggers several distinct intracellular events including calcium release or variations of the redox state that coordinate gene expression, the lack of gene expression response after EPS may be due to a lower magnitude in one particular intracellular signal or the absence of specific signals targeting a specific class of genes.

Exercise is associated with remodeling of the DNA methylation profile of several genes involved in glucose and lipid metabolism in skeletal muscle (4). Using our EPS protocol in cultured myotubes, we confirm that contraction alters DNA methylation status at promoter regions of specific genes. Depending on the type of assay used to detect DNA methylation, a loss in DNA methylation can be caused by a true demethylation or, by an additional modification on the methylated cytosine that escape detection. For example, DNA methylation capture assays relying on antibodies targeting 5-methylcytosine (MeDIP) would fail to distinguish between demethylation and hydroxylation of methylated cytosine. Bisulfite sequencing relies on the property of sodium bisulfite to convert unmodified cytosine to uracil and therefore, unlike MeDIP, can be used to detect true cytosine demethylation. Thus, in this study, we used bisulfite sequencing as a direct measure of DNA methylation. Our results provide evidence that EPS-stimulated myotube contraction induces active demethylation on the promoter of *Nr4a3, Ccnd1*, and *Ppard*. Using hydroxymethylation capture, we reveal that hydroxymethylcytosine levels are elevated immediately after EPS, with lowest levels observed 60 min thereafter, corresponding to a time point where demethylation has peaked. Active demethylation occurs through two distinct steps: the first step involves oxidative modifications of methylated cytosines to hydroxymethylcytosines by the TET enzymes and the second step corresponds directly to demethylation, where modified cytosines are replaced by cytosines by the base-excision repair machinery (31). Although we did not establish a direct relationship between methylated cytosine hydroxylation and DNA demethylation, our results support the notion that EPS contraction activates a TET enzyme and this leads to a hydroxymethylation-based demethylation of exercise-responsive genes.

Skeletal muscle contraction generates intracellular metabolites that may participate in demethylation of gene promoters (2). This hypothesis is supported by the recent observation that the intermediate metabolite of the Krebs cycle, alpha-ketoglutarate (aKG), a catalytic activator of the TET enzymes (31), is implicated in brown adipocyte differentiation through demethylation of the master regulator of brown adipocyte differentiation *Prdm16* (32). In addition, activation of the AMP kinase by the pharmacological agent AICAR increases levels of aKG and this is associated with demethylation of *Prdm16* (32). Thus, AMP kinase activation and fuel utilization during active muscle contraction could change the intracellular availability of aKG, which could in turn regulate TET-dependant DNA demethylation of exercise-responsive genes. Other intracellular metabolites could also participate in contraction-induced demethylation. For instance, intracellular availability of the antioxidant Vitamin C, an allosteric activator of TET enzymes (33), is likely to be influenced by contraction-induced changes in the redox state and may thereby modulate TET enzyme activity. Dietary interventions and metabolomic studies are warranted to assess the potential role of metabolite changes during muscle contraction and the effect of these metabolites on the epigenetic remodeling of exercise-responsive genes.

In conclusion, we have established an EPS model to further explore DNA demethylation after muscle contraction. We identified acute increase in hydroxymethylation levels followed by demethylation of the *Nr4a3* promoter and proposed a role of DNA demethylation in the control of gene expression after exercise. Understanding how exercise orchestrates gene expression changes may provide therapeutic entry points for a plethora of age-related and metabolic diseases.

## Data Availability

Bisulfite-Seq and RNA-Seq data are deposited in the NCBI Gene Expression Omnibus database with the accession number GSE87749.

## Author Contributions

RB was the guarantor of this work and, as such, had full access to all the data in the study, and took responsibility for the integrity of the data and the accuracy of the data analysis. RB and PP contributed to the study design; PP and OF contributed to data acquisition; PP, CG, OF, and RB contributed to data analysis. All the authors contributed to data interpretation and manuscript drafting and approved the final version of the manuscript.

## Conflict of Interest Statement

The authors declare that the research was conducted in the absence of any commercial or financial relationships that could be construed as a potential conflict of interest.
