# Supplementary material for: Muscle Contraction Induces Acute Hydroxymethylation of the Exercise-Responsive Gene Nr4a3
Source: Front Endocrinol (Lausanne). 2016 Dec 23;7:165. doi: 10.3389/fendo.2016.00165 (PMC5179501; doi:10.3389/fendo.2016.00165)
Supplement: Supplementary file 1 [file Table_1.DOCX]

**Supplementary Table 1:** Clinical characteristics of the participants.

| **n = 10** | **Mean** | **SD** |
| --- | --- | --- |
| Age | 22.6 | 1.6 |
| Weight (kg) | 79.2 | 14.5 |
| Height (cm) | 185 | 9 |
| Body mass index (kg/m^2^) | 23.1 | 2.8 |
| Waist circumference (cm) | 87 | 7 |
| Hip circumference (cm) | 94 | 6 |
| Waist/Hip *ratio* | 0.92 | 0.03 |
| VO_2_max (ml O_2_/min/kg) | 45.47 | 5.13 |
| Plasma glucose level (mmol/L) | 4.83 | 0.41 |
| Plasma insulin level (mmol/L) | 57.20 | 20.16 |
| HOMA-IR | 2.08 | 0.88 |
| C-reactive protein (mg/L) | 1.5 | 0.8 |
| Triglycerides (mmol/L) | 1.03 | 0.37 |
| Total cholesterol (mmol/L) | 4.29 | 0.69 |
| HDL-chol (mmol/L) | 1.38 | 0.24 |
| LDL-chol (mmol/L) | 2.34 | 0.52 |
