# Supplementary material for: Muscle Contraction Induces Acute Hydroxymethylation of the Exercise-Responsive Gene Nr4a3
Source: Front Endocrinol (Lausanne). 2016 Dec 23;7:165. doi: 10.3389/fendo.2016.00165 (PMC5179501; doi:10.3389/fendo.2016.00165)
Supplement: Supplementary file 2 [file Table_2.DOCX]

**Supplementary Table 2:** Sequences of primers used for gene expression analysis (qPCR primers) bisulfite sequencing (bisulfite primers) and hydroxymethylcytosine capture-PCR (OHCH_3_-PCR primers).

| **Gene Name** | **Forward Primer (5’-3’)** | **Reverse Primer (5’-3’)** |
| --- | --- | --- |
|  |  |  |
| **qPCR primers** |  |  |
| ***Nr4a3*** | AGCTGGGCAGAAAAGATCCC | AAGGCACTGAAGTCGATGCA |
| ***Ppard*** | CCCCGGAGCTCAATGGGGGA | GGTCCAGCAGGGAGGAAGGGG |
| ***Ccnd1*** | CTGCCGAGAAGTTGTGCATC | GCCAGGTTCCACTTGAGCTT |
| ***Ppargc1a*** | ACCAGTACAACAATGAGCCTGCGA | TCCAGTGTCTCTGTGAGAACCGC |
|  |  |  |
| **Bisulfite-primers** |  |  |
| ***Nr4a3*** | GAAGTTTTAGGGTTGGGGTT | CCTCTCCATAAAATACCTAAAATAC |
|  | GGGGTAGTTGTTGTTGTATTTATGAT | CATTACAAACCTCTAAACCAAATCC |
| ***Ppard*** | TTGTATTAGATGATTTTTATTTTTTTT | CCTAAAACCACTCCTTTAACAACC |
|  | GTTTGAGGAGAATTTAGAGGTGTA | AAAATCAACAAACTCATAAAAAAAA |
| ***Ccnd1*** | TTTGGTTAGGTTAGTTTGTTTAGTT | AAACAATAATTCCACTATAACTCC |
|  | AGGTTGGGGATTTTTTAAAGTTTAG | TACAAAAACACCCTATTCTTAAACC |
|  |  |  |
| **OHCH_3_-PCR primers** |  |  |
| ***Nr4a3*** | CTCAGGAAGTGGTGGCCG | CGCTGCCTGGGGTGC |
